# An Atypical Presentation of an Osteoid Osteoma of the Lesser Trochanter Resected via the Ludloff Approach

**DOI:** 10.7759/cureus.67811

**Published:** 2024-08-26

**Authors:** Achraf Tebbaa El Hassali, Mohammed Barrached, Adnane Lachkar, Najib Abdeljaouad, Hicham Yacoubi

**Affiliations:** 1 Orthopedics and Traumatology, Mohammed VI University Hospital, Faculty of Medicine and Pharmacy of Oujda, Mohammed I University, Oujda, MAR

**Keywords:** surgery, ludloff approach, lesser trochanter, osteoma, osteoid

## Abstract

Osteoid osteoma is a benign bone tumor that frequently affects young adults. The clinical presentation is variable, and the course can lead to spontaneous regression or persistence, necessitating medical or surgical treatment. We report the case of a young patient with an exceptional localization of an osteoid osteoma at the lesser trochanter, exhibiting atypical clinical and radiological features. The tumor was resected via the Ludloff approach. Here, we discuss our diagnostic and therapeutic approach in light of the literature.

## Introduction

Osteoid osteoma is a benign bone tumor. In its classic form, it primarily affects long bones (femur and tibia) and represents 12% of all benign tumors and approximately 3% of bone tumors [1]. It most commonly affects young adults, with a clear male predominance [1]. Management typically involves surgical excision of the tumor, with rare cases of recurrence, while spontaneous regression may occur within 6-15 years without treatment [1,2].

Here, we report the case of a young patient with an exceptional localization of osteoid osteoma at the lesser trochanter, presenting with atypical clinical and radiological features, and resected via the Ludloff approach. We discuss our diagnostic and therapeutic approach in light of the literature.

## Case presentation

A 19-year-old student had no notable personal or family medical history. The onset of his condition dated back two years, marked by the progressive appearance of pain in the left groin area during physical activity. The pain was intermittent, moderate-to-severe in intensity, without radiation or associated signs. Initially, the patient self-medicated with 3 g/day of paracetamol without significant improvement. The condition worsened, with the pain becoming constant and more intense over the past two months, in the absence of fever and with a stable general condition, prompting a consultation with orthopedic trauma.

Clinically, the patient was conscious and stable neurologically, hemodynamically, and respiratorily. Inspection of the groin did not reveal inflammatory signs or visible masses. Palpation was painful, but standing was possible. His walking was normal without limping, and his range of motion in the hip and knee was preserved. Lymph nodes were free, and the examination of the contralateral side was normal.

**Figure 1:**
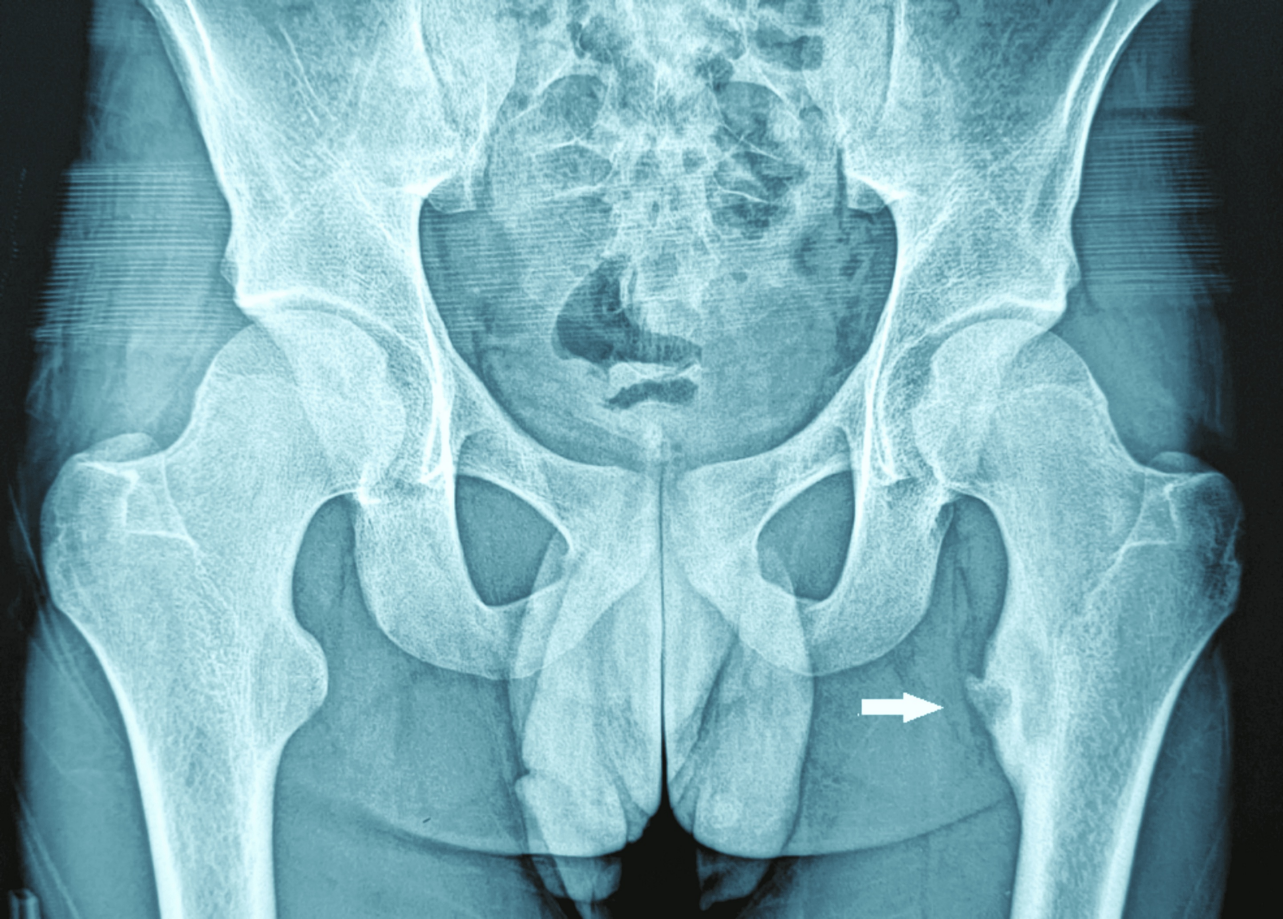
Standard radiography of the pelvis showing a lytic lesion of the left lesser trochanter with an internal cortical breach.

The patient underwent standard radiography of the pelvis and a lateral view of the left hip, showing a lytic lesion of the left lesser trochanter with regular, well-defined contours, surrounded by a rim of osteocondensation, with an internal cortical breach but without periosteal reaction or signs of soft tissue invasion (Figure 1).

**Figure 2:**
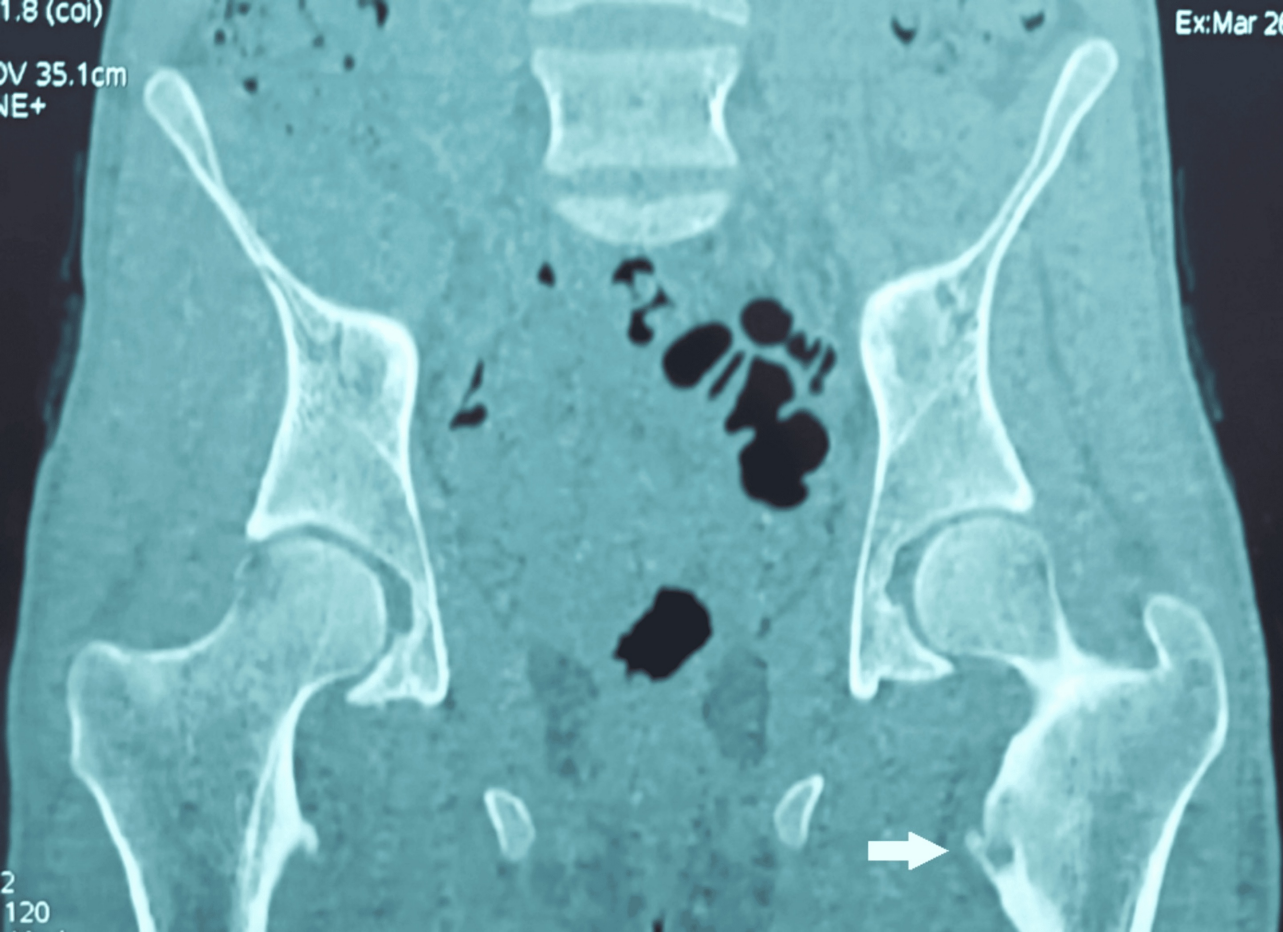
A non-contrast CT scan of the pelvis in front section showing the lesion of the left lesser trochanter.

**Figure 3:**
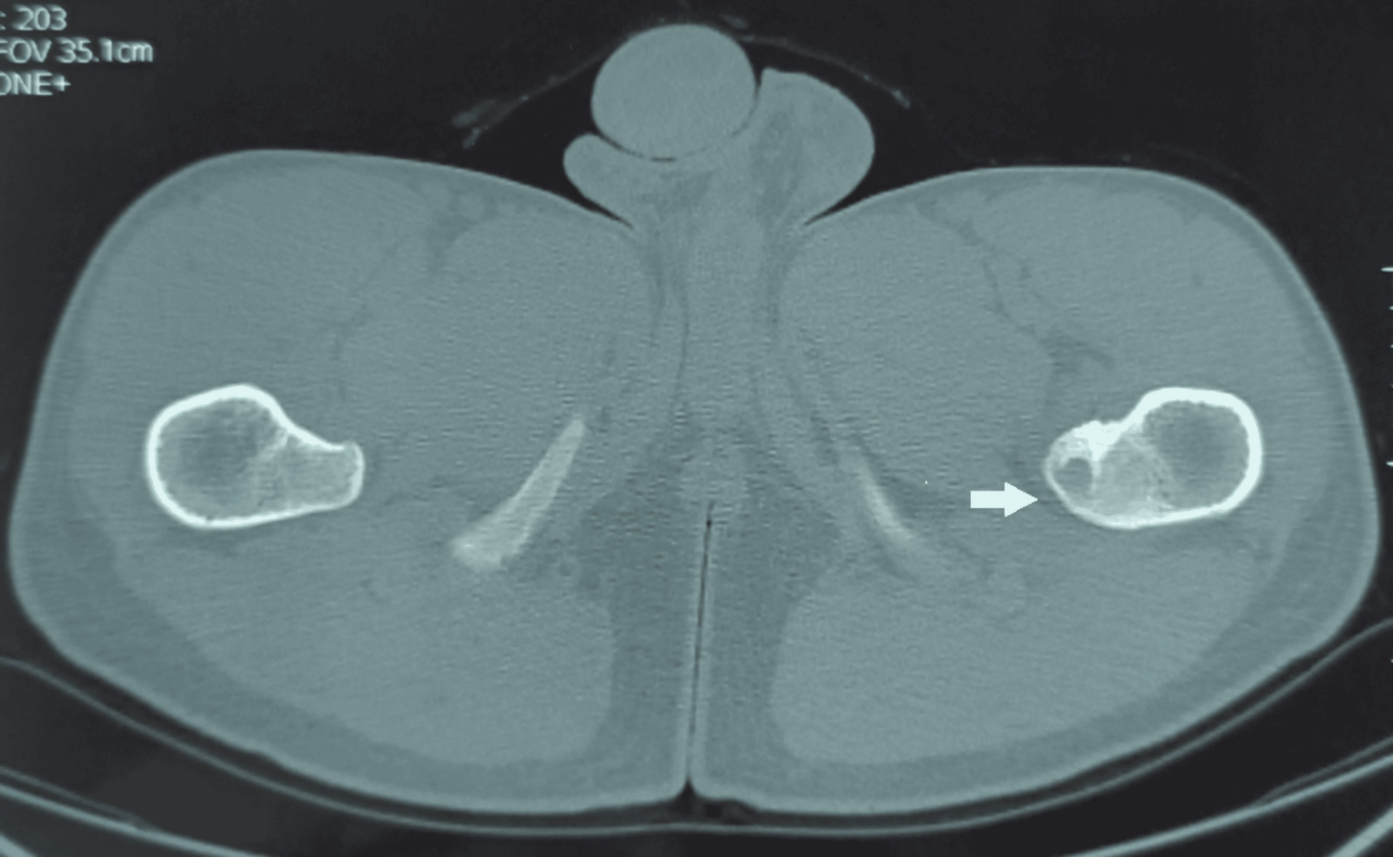
A non-contrast CT scan of the pelvis in the axial section showing the lesion of the left lesser trochanter.

A non-contrast CT scan of the pelvis revealed a spongy bone lesion of the left lesser trochanter with both osteocondensing and cystic components, as well as a cortical breach, suggestive of a small non-ossifying fibroma according to the radiologist’s interpretation (Figures 2, 3). The patient also underwent an MRI, which did not show signs of soft tissue invasion.

**Figure 4:**
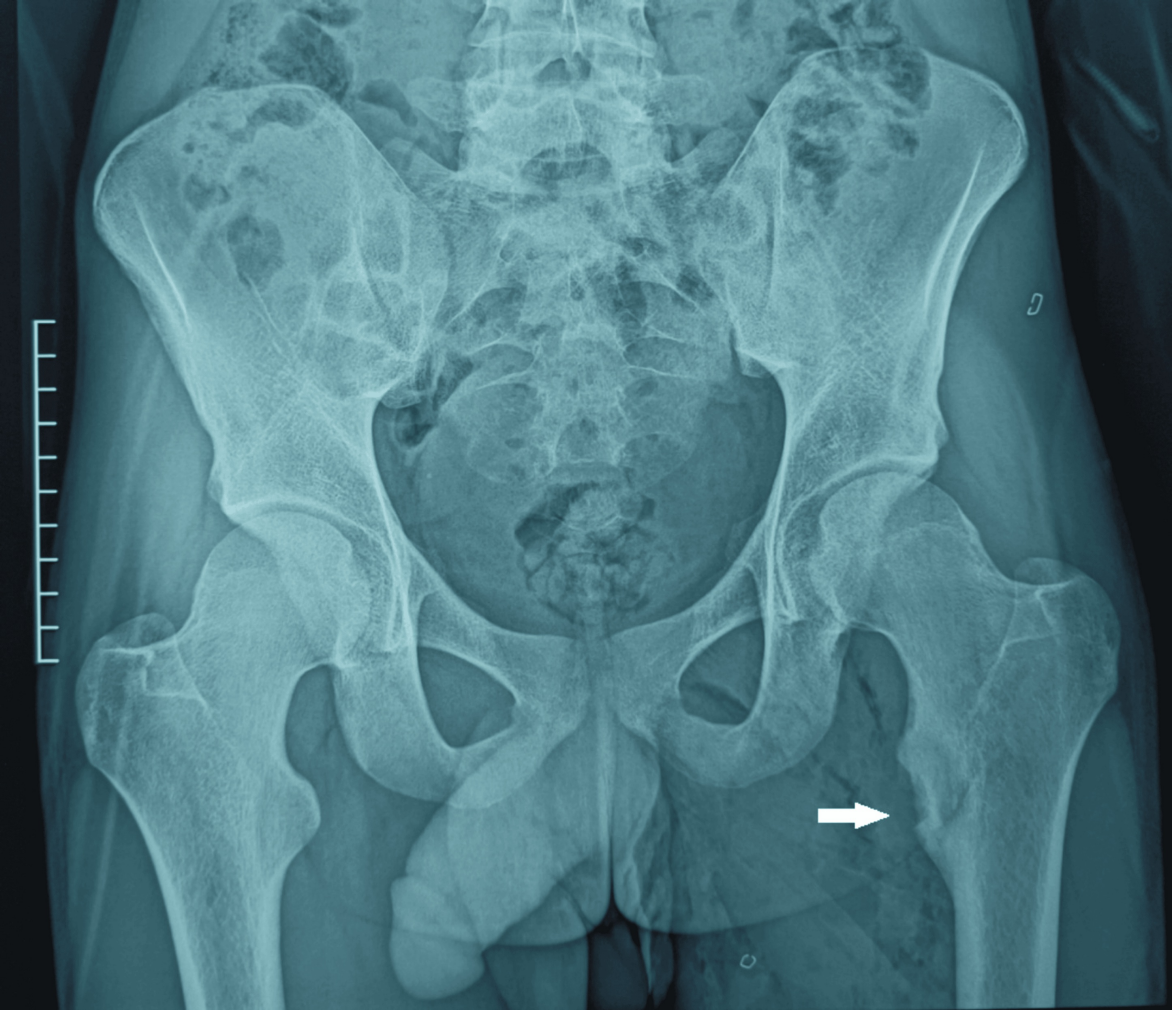
Postoperative standard radiography of the pelvis after surgical resection of the tumor.

Given the clinical and radiological suspicion of a benign bone lesion, and considering the pain reported by the patient, we decided to proceed with a surgical biopsy and excision of the lesion via the medial Ludloff approach. During surgery, we encountered a lesion typically indicative of an osteoid osteoma with the presence of a nidus. We performed its excision while preserving the insertion of the psoas muscle tendon (Figure 4).

The histopathological examination of the resected specimen confirmed the diagnosis of an osteoid osteoma. Postoperative clinical and radiological follow-up at six months showed an almost complete resolution of the pain, with preserved hip function and normal, non-limping gait.

## Discussion

Osteoid osteoma is a small, highly vascularized bone tumor [2]. In its typical form, it presents as a nidus surrounded by sclerotic bone. It consists of variable proportions of osteoid and woven bone surrounded by osteoblasts forming irregular trabeculae interspersed with osteoclasts and numerous dilated vessels [3]. The tumor does not invade adjacent bone but can induce hyperostosis or edema [3].

The clinical presentation of osteoid osteomas is highly variable and depends on the size and location of the tumor. It often presents as intermittent, progressively worsening pain, especially at night, which is alleviated by aspirin or non-steroidal anti-inflammatory drugs (NSAIDs). Painless swelling or reduced mobility has also been reported [3]. The tumor can cause synovitis and joint effusion if located near chondral structures, or painful scoliosis if located in the spine [4]. The differential diagnosis covers a broad range of conditions due to the variable presentation of osteoid osteoma, and the diagnostic process can sometimes be a challenge for clinicians [4].

The evolution of osteoid osteomas is unpredictable. Some reports suggest spontaneous regression after 6-15 years, which may be reduced with the use of aspirin and NSAIDs. Others describe several innovative therapeutic methods [5,6].

Osteoid osteomas are identifiable on standard X-rays, but sometimes CT scans or MRIs are necessary for a more precise study of the lesions. Bone scintigraphy can aid in diagnosis, while osteoarticular ultrasound has limited utility [7].

In practice, CT is the best imaging modality to detect the nidus, and MRI is the best modality to identify and study associated soft tissue and bone marrow lesions. The classic but inconsistent appearance is often described as a “target.” Rarely, the nidus is almost completely ossified and mimics a bone island or normal cortical bone. Generally, the cortical bone adjacent to the nidus is thickened. When present, the periosteal reaction is usually solid, rarely showing a multilayered appearance [7-9].

Interventional radiology offers percutaneous therapeutic techniques guided by CT, including trephine excision, cryoablation, radiofrequency ablation, and laser thermocoagulation [10].

Surgical treatment has long been the gold standard for managing osteoid osteomas, particularly when the tumor is accessible or when there is diagnostic uncertainty requiring histological confirmation. Despite technological advances, curettage remains a good therapeutic option [11].

Arthroscopic resection of an intra-articular lesion appears to yield very satisfactory results [12]. Despite surgical or percutaneous treatment, tumor recurrence occurs in 4-12% of cases. Recurrence is generally considered the result of incomplete excision, removal, or destruction. Cases of recurrence at a distance from the initial site have also been reported [11-14].

Medical management may be considered if the tumor is clinically tolerated or if other therapeutic options are difficult or risky. It relies on the use of aspirin or NSAIDs. Clinical trials are ongoing regarding bisphosphonates for this indication [15-17].

Similarly, our therapeutic approach and a series of cases surgically treated via the medial Ludloff approach have reported satisfactory postoperative results without notable complications. This approach does not preclude more radical surgery in the case of malignant histological results [18].

## Conclusions

The diagnosis of osteoid osteoma is not always straightforward. Clinical and radiological polymorphism can lead to erroneous diagnoses. The therapeutic approach has evolved from surgical resection to percutaneous therapies, and the option of medical treatment remains debated for certain specific cases.
